# The effectiveness and safety of the active form of folate on biochemical parameters in women of childbearing age: A systematic review and meta-analysis

**DOI:** 10.1097/MD.0000000000046564

**Published:** 2025-12-12

**Authors:** Min Xie, Xuemei Qing, Hailong Huang, Jing Zhang

**Affiliations:** a Department of Obstetrics and Gynecology, Chengdu Qingbaijiang District People’s Hospital, Chengdu, China; b Department of Obstetrics and Gynecology, West China Second University Hospital, Sichuan University, Chengdu, China; c The Joint Laboratory for Reproductive Medicine of Sichuan University, The Chinese University of Hong Kong, Chengdu, China; d Reproductive Endocrinology and Regulation Laboratory, West China Second University Hospital, Sichuan University, Chengdu, China.

**Keywords:** 5-methyltetrahydrofolate, active form of folate, erythrocyte folate, folic acid, homocysteine, meta-analysis, plasma folate

## Abstract

**Background:**

Low folate levels in women of childbearing age can cause various health issues. Additionally, low perinatal folate concentrations are a significant cause of neural tube defects. Currently, folic acid supplements mainly consist of folic acid and the active form of folate. Therefore, this systematic review and meta-analysis aimed to evaluate the effectiveness and safety of the active form of folate in women of childbearing age.

**Methods:**

We searched the published literature in PubMed, Medline, EMBASE, The Cochrane Library, China National Knowledge Infrastructure, and Wanfang. Randomized controlled trials (RCTs) were obtained to assess the effects of the active form of folate versus folic acid in women of childbearing age. The random or fixed effects model was used to analyze the data in meta-analysis. The results were expressed as standardized mean differences or risk ratios along with their corresponding 95% confidence intervals.

**Results:**

Eleven RCTs were identified in our systematic review. The results indicated that the active form of folate supplementation might significantly increase plasma folate (*P* = .04), increase erythrocyte folate (*P* = .01), and decrease unmetabolized folic acid (*P* < .0001). Supplementation with the active form of folate might increase the subsequent pregnancy rates (*P* = .0005) and might decrease the incidence of adverse pregnancy outcomes (*P* = .0003) in women with a history of adverse pregnancy outcomes. However, there were no significant differences in homocysteine, vitamin B_12_, and betaine. In addition, subgroup analyses showed a significant increase in plasma folate and erythrocyte folate in the active form of folate supplementation group, specifically in subgroups with dosage ≥0.4 mg, intervention of the active form of folate supplementation versus the equimolar mass of folic acid, and intervention duration ≥12 weeks.

**Conclusion:**

Active form of folate supplementation might contribute to higher plasma folate, erythrocyte folate, and subsequent pregnancy rates, lower unmetabolized folic acid, and lower incidence of adverse pregnancy outcomes in women of childbearing age. Due to the limitation in the quality of involved studies and the short duration of treatment, more RCTs with high-quality, long-term duration and pregnancy outcomes are needed for further validation.

## 1. Introduction

Folic acid, a water-soluble B vitamin, plays essential roles in various physiological processes, including nucleic acid synthesis, cell proliferation, and methylation reactions that regulate homocysteine metabolism and epigenetic modifications.^[1]^ Adequate folate status is particularly crucial for women of childbearing age, as deficiency has been linked to a spectrum of adverse health outcomes, including neurological disorders, cardiovascular disease, depression, and impaired reproductive health.^[2,3]^ The most well-established consequence of periconceptional folate deficiency is an increased risk of neural tube defects (NTDs), a class of severe congenital malformations that can result in fetal or infant mortality and permanent disability.^[4,5]^ As the second most common type of birth defect globally, NTDs affect approximately 1 in 1000 live births, accounting for nearly 3,00,000 cases worldwide annually.^[6]^ Robust evidence has demonstrated that periconceptional folic acid supplementation can significantly reduce the risk of NTDs in the developing fetus by 50% to 70%.^[5]^ Furthermore, low folate levels are associated with elevated homocysteine, an independent risk factor for various pregnancy complications, including recurrent spontaneous miscarriage. More than 20% of women with idiopathic recurrent miscarriage exhibit elevated plasma total homocysteine. Folic acid supplementation effectively lowers homocysteine levels and reduces miscarriage risk.^[7,8]^ Consequently, maintaining sufficient folic acid levels is paramount for maternal and fetal health. In 2023, the US Preventive Services Task Force recommended a daily intake of 400 to 800 μg of folic acid for those planning or capable of pregnancy.^[9,10]^ The implications of both low and high folate levels during preconception and early pregnancy are summarized in Table 1, highlighting the critical balance required for optimal reproductive outcomes.

**Table 1:** Implications of low and high folate levels in women during preconception and early pregnancy.

Folate status	Possible implications in preconception and early pregnancy
Low/deficient (e.g., RBC folate < 305 nmol/L)	• Increased risk of neural tube defects (NTDs) • Megaloblastic anemia • Elevated homocysteine level (associated with adverse pregnancy outcomes) • Potential impact on other birth defects (e.g., cardiac anomalies) • Increased risk of miscarriage
Adequate/sufficient (e.g., RBC folate ≥ 906 nmol/L)	• Significant reduction in the risk of NTDs • Prevention of folate-deficiency anemia • Support for rapid fetal growth and development • Normalization of homocysteine levels
High/supraphysiological (e.g., due to very high supplemental doses)	• Potential masking of vitamin B12 deficiency • Unmetabolized folic acid (UMFA) accumulation • Uncertain effects on other health outcomes (e.g., some studies suggested potential risks for allergies or asthma in offspring, but evidence is inconclusive) • Generally considered safe within the recommended upper limits

NTD = neural tube defect, RBC = red blood cell, UMFA = unmetabolized folic acid.

However, humans cannot synthesize folic acid endogenously, and the folic acid in natural foods is often insufficient to meet the elevated demands of pregnancy, making supplementation a public health necessity.^[11]^ The 2 primary forms of folic acid supplementation are synthetic folic acid found in fortified products and dietary supplements, and the active form of folate, which includes 5-methyltetrahydrofolate (5-MTHF), (6*S*)-5-methyltetrahydrofolate ((6*S*)-5-MTHF), L-methylfolate, tetrahydrofolates, and the salts of L-5-methyltetrahydrofolate (L-5-MTHF).^[12]^ Synthetic folic acid requires enzymatic conversion in the liver to become active 5-MTHF. Although folic acid is stable and inexpensive, its metabolism poses certain limitations: it can mask vitamin B_12_ deficiency, and high intake may lead to circulating unmetabolized folic acid (UMFA), which has been associated with potential immune modulation and other health concerns.^[13–16]^ Moreover, polymorphisms in the methylenetetrahydrofolate reductase (MTHFR) gene are highly prevalent in the general population. Common genetic variants in the MTHFR gene (e.g., C677T and A1298C) can impair the conversion of folic acid to its active form. The prevalence of these polymorphisms varies across populations, with the C677T variant occurring in 20% to 40% of US Caucasians and A1298C in 7% to 12% of individuals in North America, Australia, and Europe.^[17]^ Notably, in China, the prevalence of the homozygous C677T genotype varies regionally, as low as 9.8% in Guangdong province, compared to 34.9% in Hebei and 36.3% in southern Beijing.^[18]^ These genetic variations substantially diminish the efficacy of folic acid supplementation in a large subset of women, underscoring the need for personalized folate approaches.

In contrast, the active form of folate (5-MTHF) offers a bioidentical alternative that bypasses these metabolic limitations. It constitutes 98% of circulating plasma folate, is directly bioavailable, and does not contribute to UMFA accumulation. It is also unaffected by variations in MTHFR or dihydrofolate reductase genes. However, it is more costly than synthetic folic acid.^[19]^

Current evidence regarding the comparative effectiveness of the active form of folate versus folic acid in women of childbearing age remains inconsistent. A systematic review previously concluded that 5-MTHF supplementation was the preferred folic acid for pregnancy under specific conditions because it could be used directly and was not affected by mutations in the MTHFR gene.^[20]^ Concerns about the risk of adverse effects of UMFA from folate metabolism could also be avoided.^[21]^ However, some studies have shown no significant difference between the active form of folate and folic acid in plasma folate, erythrocyte folate, and homocysteine. There is insufficient evidence to support the superiority of the active form of folate supplementation over folic acid in this population. To resolve this uncertainty, we conducted a systematic review and meta-analysis of existing randomized controlled trials (RCTs) to evaluate the safety and effectiveness of the active form of folate in women of childbearing age. This study aims to evaluate the impact of the active form of folate supplementation on biochemical parameters, including plasma folate, erythrocyte folate, homocysteine, UMFA, vitamin B_12_, and betaine, as well as reproductive outcomes, such as subsequent pregnancy rates and the incidence of adverse pregnancy outcomes (spontaneous abortion, missed abortion, NTD, anencephaly, hydrocephalus, congenital heart disease, cleft lip and palate, multiple malformations) among women with a history of adverse pregnancy outcomes. The aim is to provide reliable guidance for clinically individualized folic acid supplementation for reproductive-age women.

## 2. Methods

This systematic review and meta-analysis was designed and conducted in accordance with a predetermined protocol according to the Cochrane Handbook’s recommendations.^[22]^ We reported the results according to the Preferred Reporting Items for Systematic Reviews and Meta-Analyses (PRISMA).^[23]^ Before data extraction, the systematic review has been registered in the PROSPERO database (CRD 42023483061).

### 2.1. Search strategies

The systematic literature search was performed in PubMed, The Cochrane Library, Embase, MEDLINE, China National Knowledge Infrastructure, and the Wanfang database from inception until January 1, 2024. No limits were applied for language and publication date. Additionally, we undertook a manual search of the reference lists of the identified papers, relevant reviews, and gray literature to identify any studies that met our selection criteria. The eligibility of documents was assessed by 2 review authors. Any disagreements were resolved by discussion with the corresponding author. The complete details of our search strategy are available (S1 File, Supplemental Digital Content, https://links.lww.com/MD/Q916).

### 2.2. Study selection criteria

We carried out the initial search, removed duplicate records, and screened the titles and abstracts for relevance. Records were then identified as included, excluded, or uncertain. In case of uncertainty, the full text was acquired to identify eligibility. Studies meeting the following criteria were included in the systematic review – participants: women of childbearing age; intervention: active form of folate supplementation separately or in combination with other drugs, compared with folic acid; outcomes: erythrocyte folate, plasma folate, homocysteine, unmetabolized folic acid, vitamin B_12_, betaine, subsequent pregnancy rates and the incidence of adverse pregnancy outcomes among women with the history of adverse pregnancy outcomes, and adverse effects; study design: RCT. Exclusion criteria were as follows: quasi-randomized trials, reviews, meta-analysis, cohort or case-control studies, case reports, animal or cell experiments; studies without explicit inclusion and exclusion criteria; and studies with unavailable data and unreported target outcomes. The identified documents were evaluated independently by 2 authors. In case of disagreement, a third author was consulted to make the final judgment.

### 2.3. Data extraction

Data extraction was conducted independently by 2 authors who comprehensively reviewed each included document. Data were cross-checked to minimize potential errors, and disagreements were handled through discussion with the corresponding author. The Cochrane guidelines were used to extract the characteristics of the studies that were included. The following information was extracted from included studies: first author’s name; study country; study publication year; participants’ characteristics, including sample size and mean age; study design and duration; characteristics of interventions, including type, and dosage; and before and after changes in outcomes of plasma folate, erythrocyte folate, hcy, UMFA, vitamin B_12_, betaine, subsequent pregnancy rates, and the incidence of adverse pregnancy outcomes among women with the history of adverse pregnancy outcomes. For continuous variables, data was extracted as mean and standard deviation for post-intervention values. mean difference, when not mentioned, was obtained by deriving it from either the standard error, interquartile range, or the 95% confidence interval. If necessary, we utilized Origin 2021 to extract data from images. We endeavored to maintain the data’s integrity, and any absent data were obtained through email or telephone communication with the authors. If no response is received, the study will be excluded.

### 2.4. Data synthesis and analysis

All related statistical analysis was conducted by using the software Review Manager 5.4. The pooled effect sizes were considered as standardized mean differences (SMDs) or risk ratios with 95% confidence intervals (95% CI). If multiple time points were reported for outcomes, the data from the final time point was used for analysis. Due to the lack of data, the minimum number of studies for the meta-analysis was decreased to 2. Heterogeneity between studies was estimated using the Cochrane *Q* statistic with its *P* value and the *I*-squared (*I*^2^) statistic (degree of heterogeneity). In each analysis, heterogeneity was presented as low (*I*^2^ < 40%), moderate (40% < *I*^2^ ≤ 70%), or high (*I*^2^ > 70%).^[24]^ The fixed-effects model was applied when *I*² < 50%. Otherwise, the random-effects model was conducted. *P* < .05 represented statistical significance. Sources of heterogeneity were explored using subgroup and sensitivity analyses. We carried out subgroup analyses based on the dosages of folic acid supplements, intervention duration (≥12 weeks or <12 weeks), and pregnancy status. Sensitivity analyses were performed by removing 1 study in each turn from the meta-analysis, with a minimum of 10 studies, to identify any significant changes in the results obtained. If heterogeneity was high, sensitivity analyses were still conducted, even if the number of studies was fewer than ten. Additionally, Egger’s test and funnel plots were conducted to examine the publication bias when >10 studies were included in the analysis.

### 2.5. Assessment of risk of bias and evidence quality

Two independent researchers conducted the quality assessment by using the Cochrane Collaboration’s risk of bias tool for RCTs.^[22]^ The analysis was carefully reviewed, and any discrepancies were resolved through consultation with a third researcher. The Cochrane risk assessment tool has been used to assess quality at the study level. Factors related to the tool included random sequence generation, allocation concealment, application of blinding method, incomplete outcome data, selective outcome reporting, and other sources of bias. Every domain was assessed and given a verdict of “yes,” “no,” or “unclear” based on The Cochrane Collaboration’s standards. We assigned 3 categories – unclear, low, and high risk of bias – for our risk-of-bias judgments.

The Grading of Recommendations, Assessment, Development, and Evaluation, a novel approach, was used to evaluate the quality level of evidence for important clinical outcomes for five domains (risk of bias, inconsistency, imprecision, indirectness, and publication bias). The quality level of evidence was downgraded by 1 or 2 levels depending on seriousness of the limitations. Evaluation of evidence quality (high, moderate, low, or very low) was determined by 2 review authors independently, with differences resolved by discussion.^[25]^

## 3. Results

### 3.1. Studies selection and the flow chart

**Figure 1.**
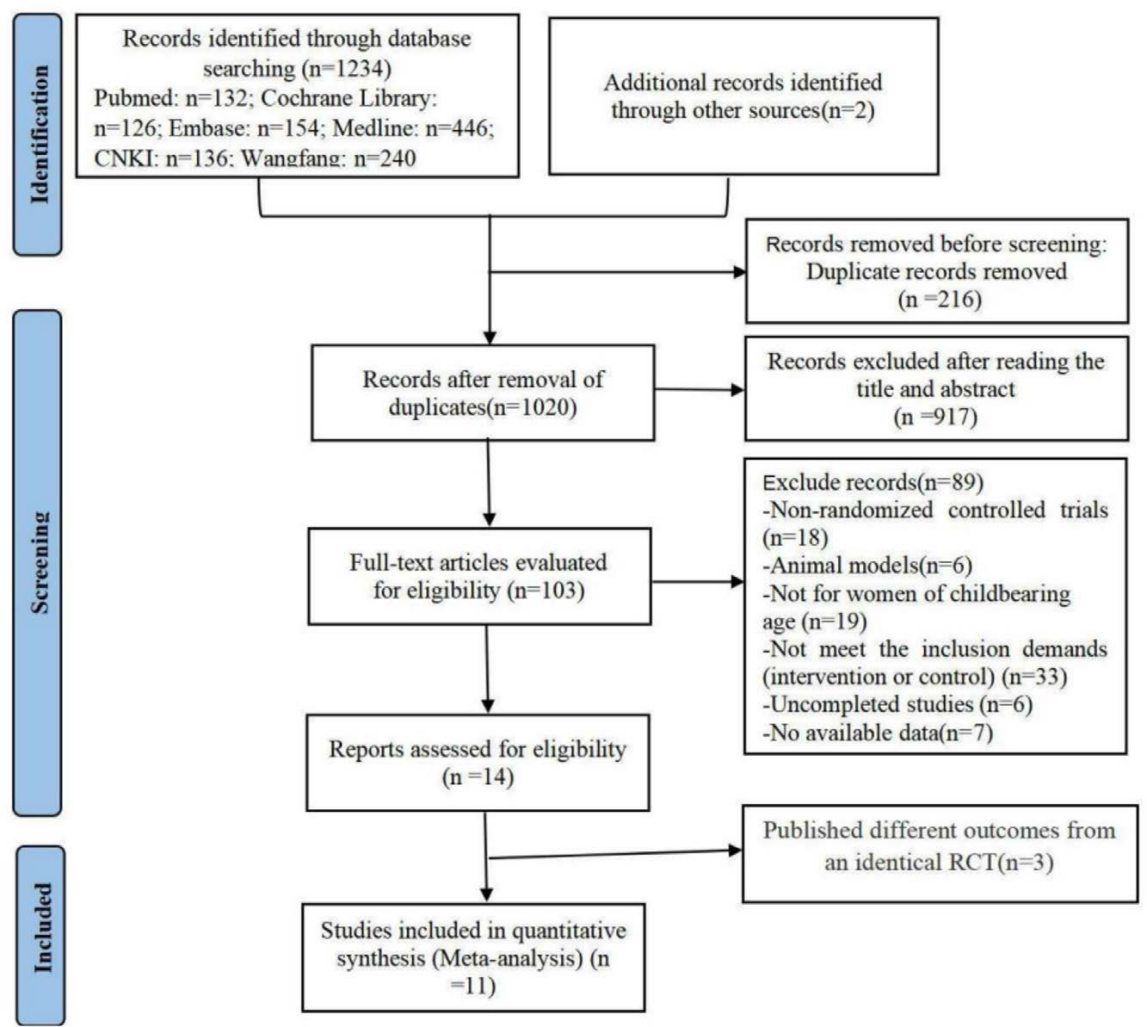
PPISMA flow diagram of the study process. PRISMA = preferred reporting items for systematic review and meta-analysis.

The preliminary search identified 1236 documents, including 132 from PubMed, 126 from Cochrane Library, 154 from Embase, 446 from Medline, 136 from China National Knowledge Infrastructure, 240 from Wanfang Database, and 2 from searching the references. After screening by Endnote, removing 216 duplicate articles, 1020 documents were assessed by screening titles and abstracts. Among them, 917 were excluded due to apparent ineligibility. One hundred three documents were selected for full-text evaluation, and 89 of these were excluded for the following reasons: non-RCTs (n = 18); animal models (n = 6); not for women of childbearing age (n = 19); intervention or control did not meet the inclusion demands (n = 33); uncompleted studies (n = 6); unavailable outcome data (n = 7). A total of 14 documents were included, some of which have reported different results but from the same randomized controlled trial.^[26–31]^ Finally, 11 RCTs (1264 participants) including 14 documents were fully synthesized in this systematic review. Details of the selection process have been shown in the PRISMA flow diagram (Fig. 1).

### 3.2. Characteristics of included studies

The summarized characteristics of the 11 RCTs including 1264 subjects are shown in Table 2. The studies were conducted in the following countries: Germany, Canada,^[26,27,32]^ China,^[33,34]^ Iran,^[35]^ New Zealand,^[36]^ Italy,^[37]^ and Malaysia.^[38]^ All of the included studies were RCTs conducted between the year of 2002 and 2023. There were 9 studies^[26–32,35–39]^ published in English and 2 studies^[33,34]^ in Chinese. Nine studies^[26–33,35,36,38,39]^ used double-blinding, while 2 RCTs^[34,37]^ were not blinded. All the included participants were females between the ages of 18 and 49 years. Folic acid supplementation was provided during the duration of pregnancy preparation and pregnancy in 5 studies.^[26,27,32–35]^ The participants were even complicated with the history of adverse pregnancy outcomes in 3 of the 5 studies.^[33–35]^ Among the other 6 included RCTs, participants were just healthy women of childbearing age but without detailed information on reproductive demand.^[28–31,36–39]^

**Table 2:** Characteristics of the included studies.

Study	Country	Publish year	Recruitment	Mean age (yr)		Sample size		Study design	Active form of folate	Folic acid	Duration (wk)	Outcomes	Adverse effects
				Active form of folate	Folic acid	Active form of folate	Folic acid						
Cochrane et al	Canada	2023	2019.09–2021.09	33 ± 3.1	33 ± 3.8	30	30	Randomized double-blind clinical trial	0·625 mg of Metafolin (Calcium salt of (6*S*)-5-MTHF) per day	0·6 mg folic acid/d	16	①②③④⑦⑧	None
Venn et al	New Zealand	2002	Unknown	Unknown		38	31	Randomized double-blind clinical trial	0.113mg (6S)-5-MTHF, calcium salt (Metafolin) per day	0.1 mg folic acid/d	24	①②	None
Lamers et al	Germany	2006	2001.12	24.2 ± 4.0	23.6 ± 3.2	36	36	Randomized double-blind clinical trial	0.416 mg [6*S*]-5-MTHF as calcium salt (Metafolin) per day	0.4 mg folic acid/d	24	①②③	None
Henderson et al	Malaysia	2018	2012.02	22.7 ± 2.89	21.3 ± 1.85	30	40	Randomized double-blind clinical trial	1.13 mg l-5-MTHF as calcium salt (Metafolin) per day	1 mg folic acid/d	12	①②③⑦⑧	None
Houghton et al	Canada	2006	2002.4–2003.12	Unknown		26	26	Randomized double-blind clinical trial	0.416 mg (6S)-5-MTHF (Metafolin) per day	0.4 mg folic acid/d	16	①②③④	None
Fohr et al	Germany	2002	Unknown	23.9 ± 3.9	23.8 ± 3.5	52	51	Randomized double-blind clinical trial	0.480 mg 5-MTHF per day	0.4 mg folic acid/d	8	①②③	None
Hekmatdoost et al	Iran	2015	2011.04–2014.09	33.6 ± 5.1	33.4 ± 4.5	110	110	Randomized double-blind clinical trial	1 mg 5-MTHF per day	1 mg folic acid/d	8	①③⑥	None
Jin et al	China	2017	2014.04–2016.04	29.57 ± 3.63	29.45 ± 3.96	122	120	Randomized double-blind clinical trial	0.4 mg methylfolate tablets per day	0.8 mg folic acid/d	Unknown	⑤⑥	None
Yunlin et al	China	2019	2015.06–2017.06	27.9 ± 2.9	28.3 ± 3.1	80	80	Randomized clinical trial	0.4 mg methylfolate tablets per day	0.8 mg folic acid/d	12	⑤⑥	None
Giunta, G et al	Italy	2018	2016.07–2016.09	31.3 ± 5.1	31.6 ± 4.6	20	20	Randomized clinical trial	LV 4 mg (calcium levofolinate pentahydrate 4 mg) per day	0.4 mg folic acid/d	8	①③	None
Diefenbach et al	Germany	2013	2006.12–2008.01	28.2 ± 5.7	27.0 ± 5.5	86	86	Randomized double-blind clinical trial	EE 0.03 mg-drospirenone 3 mg-levomefolate calcium 0.451 mg/d	EE 0.03 mg-drospirenone 3 mg + folic acid 0.4 mg/d	24	①②③	Yes

Outcomes: ① plasma folate; ② erythrocyte folate; ③ homocysteine; ④ unmetabolized folic acid; ⑤ incidence of adverse pregnancy outcomes; ⑥ the rate of pregnancies; ⑦ vitamin B12; ⑧ betaine.

(6*S*)-5-MTHF = (6*S*)-5-methyltetrahydrofolate, 5-MTHF = 5-methyltetrahydrofolate, EE = ethinylestradiol, LV = levomefolate calcium.

Various active forms of folate were included in the present review, such as 5-MTHF, 6(*S*)-5-MTHF, l-5-MTHF, levomefolate calcium, and methylfolate tablets. Metafolin (Calcium salt of (6*S*)-5-MTHF) was investigated in 9 studies,^[26–32,35–39]^ and 2 studies investigated methylfolate tablets.^[33,34]^ The intervention duration was also different among studies, ranging from 8 to 24 weeks. In all the included studies, the active form of folate supplements was compared with folic acid supplements, while 5 studies included 3 intervention arms which also compared the active form of folate supplements with placebo.^[30–32,36,38,39]^ The participants were compared before and after the intervention. Meanwhile, 6 studies conducted additional mid-term assessments.^[28,30–32,35,36,39]^ Nine studies investigated the effect of the active form of folate supplementation on plasma folate.^[26–32,35–39]^ Eight studies reported homocysteine levels,^[26–32,35,37–39]^ and 7 studies reported erythrocyte folate.^[26–32,36,38,39]^ Three studies reported the effect of the active form of folate supplementation on the rate of subsequent pregnancy in women with the history of adverse pregnancy.^[33–35]^ Two studies reported the effect of supplementation with both the active form of folate and folic acid on the incidence of adverse pregnancy outcomes among women with the history of adverse pregnancy outcomes.^[33,34]^ Two studies reported unmetabolized folic acid,^[26,27,32]^ and 2 studies reported vitamin B_12_ and betaine^[26,27,38]^ (Table 2).

### 3.3. Risk bias in included studies

**Figure 2.**
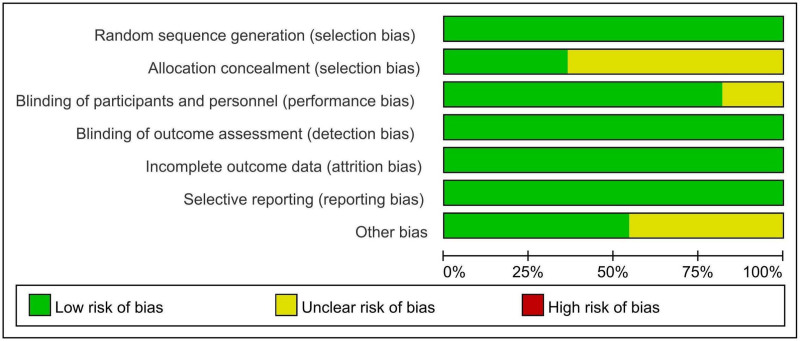
Overall risk of bias assessment.

**Figure 3.**
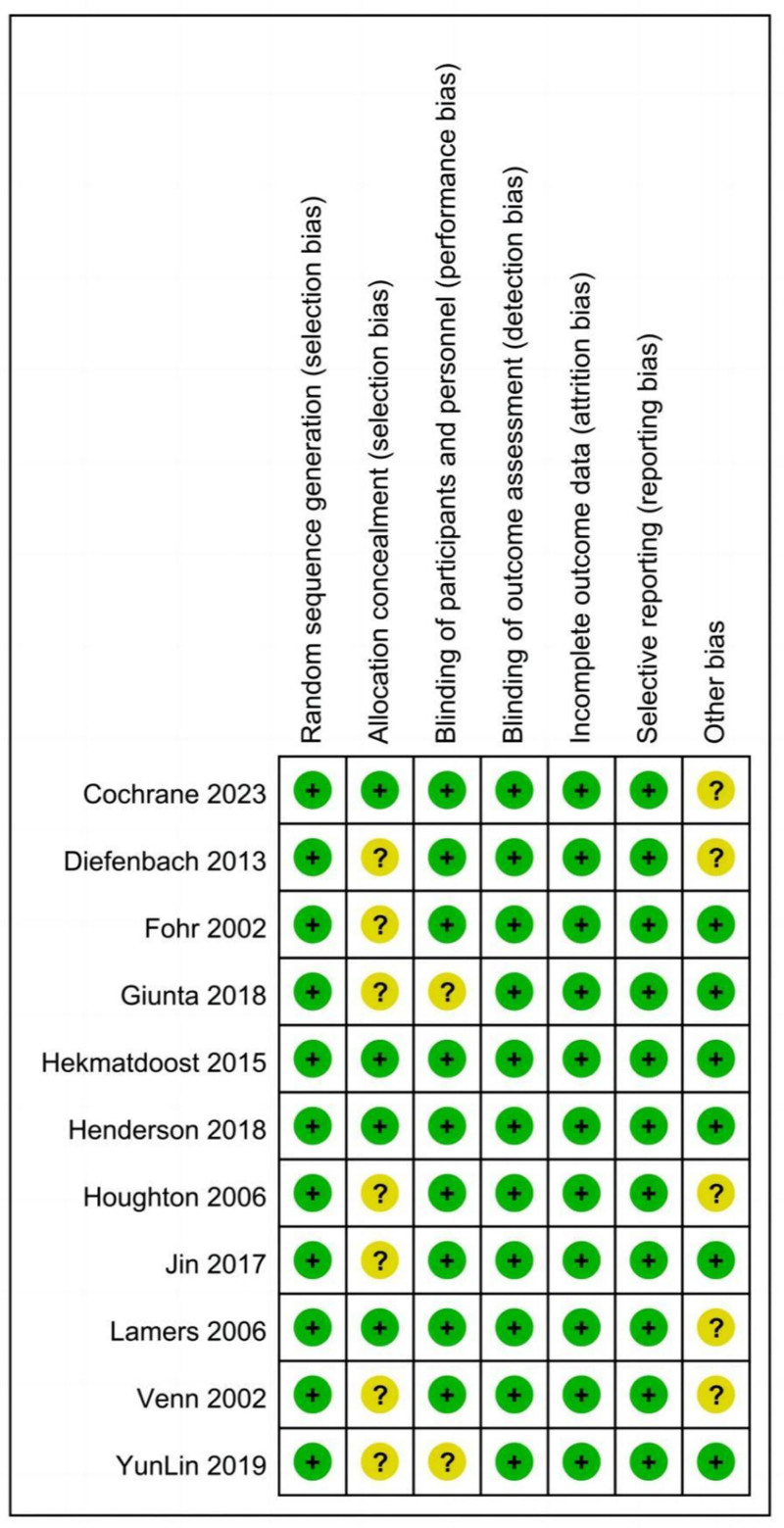
Risk of bias summary for randomized controlled trails.

Figures 2 and 3 summarize the risk bias of the included studies based on different quality domains using the Cochrane risk of bias tool. All studies reported adequate randomized sequence generation. All studies had unclear risk of bias in allocation concealment except 3.^[26,27,35,38]^ These 3 studies had a low risk of bias in allocation concealment. Participants and personnel were blinded except for 2.^[34,37]^ None of the trials reported whether the outcome assessors were blinded or not. However, the lack of blinding among outcome assessors does not affect the outcome measurement of objective serum indexes, and the risk was low. There was no selective reporting bias in any of the studies. Furthermore, the baseline characteristics of all included studies were balanced. Five studies were assessed as having unclear risk of other bias due to manufacturer sponsorship,^[26–32,36]^ while the remaining studies were assessed as having low risk of bias.

### 3.4. Data synthesis and meta-analysis

#### 3.4.1. Plasma folate

**Figure 4.**
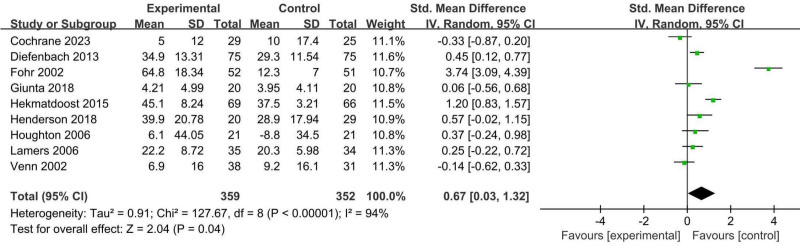
Forest plot of the analysis of plasma folate.

Nine studies (711 patients) were analyzed to explore the effect of the active form of folate supplementation on plasma folate.^[27,28,31,32,35–39]^ The results indicated a significant increase in the level of plasma folate after being supplied with the active form of folate supplementation compared to folic acid supplementation (SMD 0.67, 95% CI: 0.03–1.32, *P* = .04, *I*^2^ = 94%, Fig. 4). The heterogeneity was high among studies. Furthermore, subgroup analyses were conducted based on different dosages of the active form of folate, duration of the intervention, and pregnancy status.

Subgroup analyses indicated that the finding remained consistent across subgroups: the active form of folate supplementation dosage ≥0.4 mg (SMD 0.78, 95% CI: 0.07–1.48, *P* = .03, *I*^2^ = 94%, Table 3), the intervention of active form of folate supplementation versus the equimolar mass of folic acid supplementation (SMD 0.02, 95% CI: 0.04–0.42, *P* = .08, *I*^2^ = 49%, Table 3), and intervention duration ≥12 weeks (SMD 0.02, 95% CI: 0.04–0.42, *P* = .08, *I*^2^ = 49%, Table 3). However, the plasma folate was not different after subgroup analysis based on pregnancy status (Table 3). Meta analysis showed that the active form of folate supplementation dosage and the duration of intervention might be sources of heterogeneity (Table 3).

**Table 3:** Subgroup analyses of plasma folate, erythrocyte folate, and homocysteine.

Outcomes	Subgroup factors	Study number	Participants	Heterogeneity		Effect model	Meta analysis	
				*P* value	*I*^2^(%)		Relative effect (95% CI)	*P* value
Plasma folate	Dosages of active form folate							
	<0.4 mg	1	67	NA	NA	NA	−0.14 (−0.62, 0.33)	.56
	≥0.4 mg	8	642	<.00001	94	Random	0.78 (0.07, 1.48)	.03
	Equimolar mass	6	433	.08	49	Fixed	0.23 (0.04, 0.42)	.02
	Different molar masses	3	278	<.00001	97	Random	1.66 (−0.16, 3.48)	.07
	Study duration							
	≥12 wk	6	433	.08	49	Fixed	0.23 (0.04, 0.42)	.02
	<12 wk	3	278	<.00001	97	Random	1.66 (−0.16, 3.48)	.07
	Pregnancy status							
	Pregnancy	3	231	<.0001	91	Random	0.43 (−0.53, 1.39)	.38
	Non-pregnancy	6	480	<.00001	95	Random	0.80 (−0.12, 1.73)	.09
Erythrocyte folate	Dosages active form folate							
	<0.4 mg	1	69	NA	NA	NA	−0.06 (−0.54, 0.41)	.79
	≥0.4 mg	6	467	.004	71	Random	0.35 (0.03, 0.67)	.006
	<0.4 mg	1	69	NA	NA	NA	−0.06 (−0.54, 0.41)	.79
	0.4–0.8 mg	5	418	.01	69	Random	0.41 (0.04, 0.78)	.03
	>0.8 mg	1	49	NA	NA	NA	1.07 (0.46, 1.68)	.0006
	Equimolar mass	6	433	.002	73	Random	0.49 (0.10, 0.88)	.01
	Different molar masses	1	103	NA	NA	NA	0.10 (−0.29, 0.49)	.62
	Equimolar 0.4 mgFA	3	261	.57	0	Fixed	0.68 (0.43, 0.93)	<.00001
	Unequal molar mass of 0.4 mg FA	4	275	.01	73	Random	0.20 (−0.28, 0.68)	.42
	Study duration							
	≥12 wk	6	433	.002	73	Random	0.49 (0.10, 0.88)	.01
	<12 wk	1	103	NA	NA	NA	0.10 (−0.29, 0.49)	.62
	Pregnancy status							
	Pregnancy	2	96	.02	81	Random	0.26 (−0.69, 1.22)	.59
	Non-pregnancy	5	440	.005	73	Random	0.48 (0.10, 0.87)	.01
Homocysteine	Dosages active form folate							
	<0.4 mg	0	NA	NA	NA	NA	NA	NA
	≥0.4 mg	8	570	.06	49	Fixed	−0.01 (0.18 ,0.15)	.90
	Equimolar mass	5	364	.69	0	Fixed	−0.15 (−0.36, 0.05)	.15
	Different molar masses	3	206	.04	68	Random	0.16 (−0.35, 0.67)	.55
	Study duration							
	≥12 wk	5	364	.69	0	Fixed	−0.15 (−0.36, 0.05)	.15
	<12 wk	3	206	.04	68	Random	0.16 (−0.35, 0.67)	.55
	Pregnancy status							
	Pregnancy	3	159	.55	0	Fixed	−0.22 (−0.53, 0.10)	.18
	Non-pregnancy	5	411	.04	61	Random	−0.05 (−0.27, 0.38)	.76

Equimolar mass, the molar mass of the active form of folate is equivalent to that of folic acid; different molar masses, the molar mass of the active form of folate differs from that of folic acid.

CI = confidence interval, NA = not applicable, FA = folic acid.

The evaluation of the evidence supporting the results suggests that the findings regarding the impact of the active form of folate supplements on plasma folate levels are moderately reliable (Table 4).

**Table 4:** The evidence quality of active form of folate for childbearing age women.

Active form of folate versus folic acid						
Patient or population: childbearing age women						
Setting: childbearing age women						
Intervention: Active form of folate						
Outcomes	Illustrative comparative risks* (95% CI)		Relative effect (95% Cl)	Number of participants (studies)	Quality of the evidence (GRADE)	Comments
	Assumed risk control	Corresponding risk active form of folate				
Plasma folate		The mean plasma folate in the intervention groups was **0.67 standard deviations higher** (0.02–1.32 higher)		711 (9 studies)	⊕⊕⊕Θ moderate	SMD 0.67 (0.02–1.32)
Erythrocyte folate		The mean erythrocyte folate in the intervention groups was **0.29 standard deviations higher** (0.01 lower–0.59 higher)		532 (7 studies)	⊕⊕⊕Θ moderate	SMD 0.29 (−0.01 to 0.59)
Homocysteine		The mean homocysteine in the intervention groups was **0.01 standard deviations lower** (0.18 lower–0.16 higher)		570 (8 studies)	⊕⊕⊕Θ moderate	SMD −0.01 (−0.18 to 0.16)
Unmetabolised folic acid		The mean unmetabolised folic acid in the intervention groups was **1 standard deviations lower** (1.95–0.04 lower)		50 (2 studies)	⊕⊕⊕Θ low	SMD −1 (−1.95 to − 0.04)
Vitamin B_12_		The mean Vitamin B_12_ in the intervention groups was **0.26 standard deviations higher** (0.65 lower–0.14 higher)		101 (2 studies)	⊕⊕ΘΘ low	SMD −0.26 (−0.65 to 0.14)
Betaine		The mean betaine in the intervention groups was **0.21 standard deviations higher** (0.2 lower–0.62 higher)		97 (2 studies)	⊕⊕ΘΘ high	SMD 0.21 (−0.2 to 0.62)
The incidence of adverse pregnancy outcomes	Study population		RR 0.2 (0.09–0.48)	402 (2 studies)	⊕⊕⊕⊕ moderate	
	146 per 1000	29 per 1000 (13–70)				
Subsequent pregnancy rates	Study population		RR 1.21 (1.09–1.35)	620 (2 studies)	⊕⊕⊕Θ moderate	
	625 per 1000	756 per 1000 (681–843)				

GRADE Working Group grades of evidence.

High quality: Further research is very unlikely to change our confidence in the estimate of effect.

Moderate quality: Further research is likely to have an important impact on our confidence in the estimate of effect and may change the estimate.

Low quality: Further research is very likely to have an important impact on our confidence in the estimate of effect and is likely to change the estimate.

Very low quality: We are very uncertain about the estimate.

CI = confidence interval, RR = risk ratios, SMD = standardized mean differences.

* The basis for the assumed risk (e.g., the median control group risk across studies) is provided in footnotes. The corresponding risk (and its 95% CI) is based on the assumed risk in the comparison group and the relative effect of the intervention (and its 95% CI).

#### 3.4.2. Erythrocyte folate

**Figure 5.**
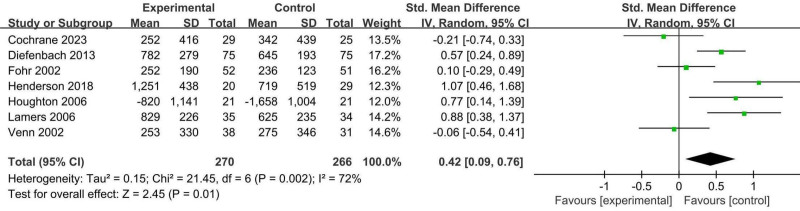
Forest plot of the analysis of erythrocyte folate.

Erythrocyte folate was assessed in 7 RCTs with 536 participants, including 270 participants in the active form of folate supplementation group and 266 participants in the folic acid supplementation group.^[27,28,30,32,36,38,39]^ The random effects model was used in the meta-analysis, and results showed that supplementation with the active form of folate significantly increased erythrocyte folate concentrations compared to supplementation with folic acid (SMD 0.42, 95% CI: 0.09–0.76, *P* = .01, *I*^2^ = 72%; Fig. 5).

Heterogeneity remained high. Subgroup analyses were conducted based on differences in supplement dosage, duration of intervention, and pregnancy status using random-effects modeling.

According to the different dosages of the active form of folate supplementation, there are 2 subgroups. Subgroup analyses revealed that higher supplemental dosages were more effective in increasing erythrocyte folate levels compared to the low-dosage (0.113 mg (6*S*)-5-MTHF/d) subgroup (SMD 0.51, 95% CI: 0.15–0.87, *P* = .006, *I*^2^ = 71%, Table 3). In subgroup analyses based on interventions of the active form of folate supplementation versus the equimolar mass of folic acid (SMD 0.49, 95% CI: 0.10–0.88, *P* = .01, *I*^2^ = 73%, Table 3), intervention duration ≥ 12 weeks (SMD 0.49, 95% CI: 0.10–0.88, *P* = .01, *I*^2^ = 73%, Table 3), and supplementation with the active form of folate in nonpregnant women of childbearing age (SMD 0.48, 95% CI: 0.10–0.87, *P* = .01, *I*^2^ = 73%, Table 3), supplementation with the active form of folate resulted in a more pronounced increase in erythrocyte folate. Furthermore, subgroup analysis of intervention showed a greater increase in erythrocyte folate after supplementation with folic acid 0.4 mg equimolar mass of the active form of folate (SMD 0.68, 95% CI: 0.43–0.93, *P* < .00001, *I*^2^ = 0%, Table 3), and the heterogeneity notably disappeared. However, the dosage of the active form of folate supplements might be a source of heterogeneity. Additionally, the meta-analysis revealed that the intervention duration and pregnancy status were not significant sources of heterogeneity (Table 3).

The evidence suggests that findings on the effects of the active form of folate supplements on erythrocyte folate levels are moderately reliable (Table 4).

#### 3.4.3. Homocysteine

**Figure 6.**
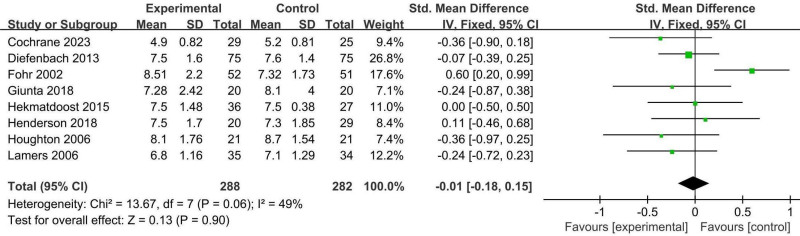
Forest plot of the analysis of homocysteine.

A meta-analysis of 8 trials (570 individuals)^[26,27,29,31,32,35,37–39]^ was conducted. Pooled results from the fixed-effects model indicated that no significant difference was found between the active form of folate supplementation group and the folic acid supplementation group in terms of lowering homocysteine (SMD −0.01, 95% CI: −0.18 to 0.15, *P* = .90, *I*^2^ = 49%, Fig. 6). No significant difference was found in the effects of various doses of active folic acid supplements, different intervention times, or pregnancy status on homocysteine levels (Table 3).

The quality of evidence for the indicated results was assessed, and it was found to have moderate confidence regarding the effect of supplementing with the active form of folic acid on homocysteine (Table 4).

#### 3.4.4. Unmetabolised folic acid

**Figure 7.**
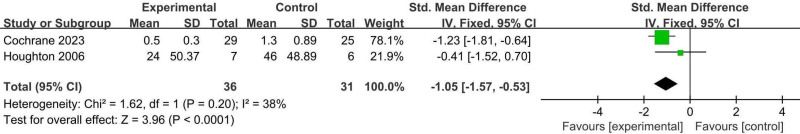
Forest plot of the analysis of unmetabolized folic acid.

Two studies^[27,32]^ containing 67 participants (36 of them in the treatment group, 31 in the control group) were included to analyze the effects of the active form of folate supplementation on unmetabolised folic acid. The level of UMFA in the active form of folate supplementation group was lower than that in the folic acid supplementation group (SMD −1.05, 95% CI: −1.57 to −0.53, *P* < .0001, *I*^2^ = 38%, Fig. 7).

One study^[39]^ reported lower concentrations of UMFA in the group that received 5-MTHF supplementation. However, the data was unavailable.

#### 3.4.5. *Vitamin B*_*12*_

The meta-analysis pooled 2 studies with a total of 101 participants.^[27,38]^ One study intervention lasted 12 weeks, and the other lasted 16 weeks. The analysis concluded that supplementation with the active form of folate had no significant effect on vitamin B_12_ levels in women compared to the folic acid supplementation group (SMD −0.26, 95% CI: −0.65 to 0.14, *P* = .21, *I*^2^ = 38%, Table 5).

**Table 5:** Active form of folate compared with folic acid for women of childbearing age.

Outcome	Study number	Heterogeneity		Effect model	Meta analysis	
		*P* value	*I* ^2^		Relative effect (95% CI)	*P* value
Plasma folate	9	<.00001	94%	Random	0.67 (0.03, 1.32)^a^	.04
Erythrocyte folate	7	.002	72%	Random	0.42 (0.09, 0.76)^a^	.01
Homocysteine	8	.06	49%	Fixed	−0.01 (−0.18, 0.15)^a^	.90
Unmetabolised folic acid	2	.20	38%	Fixed	−1.05 (−1.57, −0.53)^a^	<.0001
Vitamin B_12_	2	.20	38%	Fixed	−0.26 (−0.65, 0.14)^a^	.21
Betaine	2	.67	0%	Fixed	0.21 (−0.19, 0.61)^a^	.30
Adverse outcomes in subsequent pregnancies	2	.16	49%	Fixed	0.20 (0.09, 0.48)^b^	.0003
Subsequent pregnancy rates	3	.03	71%	Fixed	1.21 (1.09, 1.35)^b^	.0005

a SMD = standardized mean differences.

b RR = risk ratios.

#### 3.4.6. Betaine

Data were extracted from 2 studies with 101 patients.^[27,38]^ We found no apparent difference of betaine following administration of the active form of folate supplementation and folic acid (SMD 0.21, 95% CI: −0.19 to 0.61, *P* = .30, *I*^2^ = 0%, Table 5).

#### 3.4.7. Adverse pregnancy outcomes

Two studies,^[33,34]^ involving 402 participants, evaluated the effect of folic acid versus active form of folate supplementation on adverse pregnancy outcomes in women with a history of adverse pregnancy outcomes. The meta-analysis showed that an active form of folate supplementation might reduce the incidence of adverse pregnancy outcomes. It may be a safer and more effective treatment for women with a history of adverse pregnancy outcomes (RR 0.2, 95% CI: 0.09–0.48, *P* = .0003, *I*^2^ = 49%, Table 5).

#### 3.4.8. Subsequent pregnancy rates

Based on data from 3 studies involving a total of 620 participants,^[33–35]^ among women with the history of adverse pregnancy outcomes, the subsequent pregnancy rates were higher in the group that received the active form of folate supplementation compared with the group that received folic acid supplementation (RR 1.21, 95% CI: 1.09–1.35, *P* = .0005, *I*^2^ = 71%, Table 5). However, the heterogeneity was high among studies.

### 3.5. Adverse effects

One study^[28]^ included in the meta-analysis reported adverse effects, while the other studies did not. This study found similar rates of adverse events between the levomefolate calcium group and the folic acid group. Furthermore, the adverse events were unrelated to the study drug. Supplementation with the active form of folate may be a safe treatment.

### 3.6. Sensitivity analysis

The meta-analysis found significant heterogeneity among studies reporting plasma folate, and erythrocyte folate. Sensitivity analyses were performed to explore potential causes, but no significant change in the combined standardized mean differences was observed when each single study was excluded. The results of the current meta-analysis are robust and not influenced by the quality of the studies included (S2 File, Supplemental Digital Content, https://links.lww.com/MD/Q916).

## 4. Discussion

This systematic review and meta-analysis compares the efficacy and safety of the active form of folate to folic acid on plasma folate, erythrocyte folate, homocysteine, unmetabolized folic acid, vitamin B_12_, betaine, subsequent pregnancy rates, and the incidence of adverse pregnancy outcomes in women of childbearing age. The results showed that the active form of folate supplementation might result in a more significant increase in plasma folate, erythrocyte folate, and a decrease in plasma unmetabolized folic acid. Women with a history of adverse pregnancy outcomes might have a lower incidence of adverse pregnancy outcomes and higher subsequent pregnancy rates after taking an active form of folate. However, there was no adequate evidence to suggest that the active form of folate had a more favorable effect on homocysteine levels, vitamin B_12_, and betaine.

The dosage and duration of the active form of folate supplementation were very different among the included studies. Subgroup analyses showed that different dosages of the active form of folate had different effects on plasma folate and erythrocyte folate. An interesting finding was that both plasma folate and erythrocyte folate were significantly increased at high dosages of the active form of folate supplementation (≥0.4 mg) as compared with folic acid. Furthermore, the active form of folate supplementation was found to favor elevations in plasma folate and erythrocyte folate compared to supplementation with an equimolar mass of folic acid. In addition, the active form of folate supplementation group showed more significant increases in plasma folate and erythrocyte folate levels when supplemented for more than 12 weeks.

Low folate status has been associated with various birth defects, such as NTDs,^[40]^ low infant birth weight,^[41]^ and cleft palate.^[42]^ Some patients with unexplained infertility also have low folate levels.^[3,43]^ Additionally, the requirement for folic acid increases during pregnancy. Sufficient folic acid intake is crucial for normal fetal growth and development. Furthermore, folic acid enhances growth and repair mechanisms even in adulthood.^[44]^ Therefore, maintaining the correct level of folic acid in the body is of paramount importance for women of reproductive age.

Our meta-analysis indicates that the active form of folate supplementation might lead to higher plasma folate and erythrocyte folate concentrations compared to folic acid supplementation. This finding is consistent with the studies conducted by Obeid et al.^[45]^ Furthermore, Obeid et al^[45]^ also reported that the maximum plasma concentrations were achieved in a shorter period. This difference may be attributed to the fact that the active form of folate is biologically available and can be directly utilized by the body, while folic acid requires processing through the liver to form 5-tetrahydrofolate.^[46]^ The active form of folate is pharmacokinetically superior to folic acid.

Our subgroup analyses revealed no significant difference in plasma folate and homocysteine levels between the active form of folate and folic acid supplementation during pregnancy and non-pregnancy. However, supplementation with the active form of folate during non-pregnancy significantly increased erythrocyte folate levels compared to folic acid supplementation. This difference may be attributed to the varying bioavailability of folic acid during pregnancy and non-pregnancy.^[45,47]^ Additionally, subgroup analyses indicated that the active form of folate supplementation had a significant impact on the increase in plasma folate and erythrocyte folate compared to folic acid after at least 12 weeks of intervention. This finding may contrast with the results of Lamers et al^[30]^ showing that plasma folate concentrations reach a plateau after 12 weeks of folate supplementation, regardless of the form and dosage of folate supplementation. Additionally, the loss of folic acid during erythrocyte denaturation significantly increases plasma folate concentrations.

The objective of supplementing women of childbearing age with folic acid is to achieve protective erythrocyte folate concentrations (>906 nmol/L) and reduce the incidence of certain birth defects.^[48]^ In a case-control study conducted in Ireland, it was found that there is a negative association between maternal plasma and red cell folate concentrations and the risk of NTD.^[49]^ Our meta-analysis found that plasma folate and erythrocyte folate significantly increased after folic acid supplementation. However, supplementation with the active form of folate had a more significant effect on plasma folate and erythrocyte folate. This statement is consistent with the findings of Troesch et al.^[50]^ Troesch et al conducted a randomized, double-blind, controlled trial conducted on infants. The study found that the group receiving L-5-MTHF had higher erythrocyte folate concentrations at follow-up compared to the group receiving supplemental folate. Subgroup analyses showed significant differences in erythrocyte folate increases at active form of folate supplementation dosages >0.4 mg and folic acid. In contrast, Venn et al^[36]^ did not observe a significant difference. This lack of difference may be attributed to the low dosage of folic acid supplementation, resulting in erythrocyte folate concentrations below 906 nmol/L, even when plasma folate levels plateau. However, it is recommended to avoid consuming large amounts of any form of folic acid during pregnancy due to potential adverse effects shown in animal studies.^[51]^ Further clinical trials are needed to examine the long-term health effects of folic acid intake during pregnancy. However, it has been shown in previous studies that erythrocyte folate concentrations do not reach a steady state even after 24 weeks. Therefore, a longer period of time is required to assess the maximum effect of folate supplementation on erythrocyte folate accumulation.^[52]^

High levels of homocysteine have been linked to low birth weight, preterm labor, preeclampsia, and NTDs.^[53]^ Folic acid, either alone or in combination with B_6_ and/or B_12_ vitamins, is an effective treatment to reduce homocysteine plasma concentrations, even in individuals without clinically significant vitamin deficiencies.^[54]^ Our meta-analysis found no significant difference between active form of folate and folic acid in reducing homocysteine levels. This is consistent with the study conducted by Zappacosta et al Both folic acid and [6*S*]-5-MTHF (at a dosage of 200 Lg/d) resulted in a similar reduction of homocysteine concentrations after 13 weeks of treatment.^[55]^ In the study by Venn et al,^[56]^ it is important to note that the homocysteine lowering effect of [6*S*]-5-MTHF or folic acid (100 μg) was most significant in subjects with the highest baseline homocysteine concentrations. The reduction was up to 14% for folic acid and 20% for [6*S*]-5-MTHF. Genetic variations in folate metabolism may impact outcomes.

Unlike active form of folate, folic acid needs to be reduced and replaced by carbon residues before it can enter the circulation as 5-MTHF. This process involves 5,10-methylenetetrahydrofolate reductase. There is limited data on the effect of [6*S*]-5-MTHF or folic acid on plasma folate in the TT genotype. A study by Prinz-Langenohl et al demonstrated that [6*S*]-5-MTHF was more effective than folic acid in increasing total plasma folate concentrations. In subjects with the TT and CC genotypes and the MTHFR 677 C→T mutation, short-term courses of equimolar test dosages showed that the area under the curve of plasma folate concentration versus time was significantly higher after administration of [6*S*]-5-MTHF than that of folic acid. Furthermore, the variance in time required to reach maximum concentration after folic acid application between TT and CC genotypes may be attributed to a reduction in 5,10-methylenetetrahydrofolate reductase activity.^[57]^ In a similar study, Willems et al^[58]^ demonstrated that 5-MTHF was more bioavailable than folic acid in elderly cardiovascular patients with TT and CC genotypes.

Unmetabolized folic acid in the blood may interfere with the metabolism, transport, and function of natural folate in the body.^[59]^ Additionally, it may mask unrecognized hematological manifestations of vitamin B_12_ deficiency, which can predispose an individual to irreversible neurological damage.^[60]^ Animal studies have shown that unmetabolized folic acid inhibits purine synthesis and the formation of 5-MTHF, which is required for DNA synthesis and methylation. Additionally, folic acid inhibits angiogenesis, interferes with normal development, and causes early cardiovascular and embryonic developmental defects. Extended use of folic acid supplements, resulting in a significant increase in unmetabolized folic acid, may elevate the risk of pregnancy-associated hypertension and preeclampsia.^[45,61]^ This statement is in line with the findings of our meta-analysis. Furthermore, it is noteworthy that MTHF-Ca did not exhibit any inhibitory effects on early embryonic cardiovascular development. As MTHF-Ca is known to be free of potential adverse effects, this natural and biologically active form of folic acid appears to be more appropriate for high-dosage folic acid supplementation.

Although this meta-analysis highlights the advantages of the active form of folate over folic acid, it is important to notice its limitations. The included studies were generally short-term, lasting between 8 and 24 weeks. Clinical outcomes may vary depending on the duration of the intervention, the dosage of folic acid supplementation, the type of participant, regional differences, and dietary habits. These factors may contribute to heterogeneity between studies. Additionally, the experimental and control groups included different genotypes of folate metabolism. It is important to note that patients with different genotypes may exhibit varying levels of folate in their bodies after folate supplementation. However, the original study did not investigate the impact of this factor on the results. Although some studies did not find differences in baseline comparisons between groups, these may still influence clinical outcomes. In addition, some of the data from the original studies were extracted by reading the graphs, and this is also a possible source of bias. Publication bias was not assessed in all outcomes because the number of included studies was <10. While most of the included studies were of high quality, the number of articles and participants was insufficient. Further investigation is needed to determine whether there are differences in the effects of supplementation with different forms of folic acid during pregnancy compared to non-pregnancy.

A growing body of evidence highlights the benefits of an active form of folate for women of childbearing age, such as increased bioavailability, production of less unmetabolized folic acid, and independence from folate metabolism genotypes. Our study also demonstrated the beneficial effects of active folic acid. However, there were differences in the dosage of different folic acid supplements, duration of supplementation, and the metabolism of folic acid during pregnancy and non-pregnancy. Therefore, more large-scale RCTs are needed to assess the true impact of an active form of folate in women of childbearing age. Particular attention should be given to subpopulations with comorbidities such as thrombophilia, where hyperhomocysteinemia – a known prothrombotic risk factor – may be mitigated through optimized folate supplementation. Establishing the optimal form and dosage of folate for high-risk individuals represents an urgent clinical research priority. Based on our analysis, which utilized (6*S*)-5-methyltetrahydrofolate (5-MTHF) at daily doses of 400 to 1000 μg, a daily supplement of 400 to 800 μg of the active form of folate during preconception and the first trimester is suggested as a viable and effective strategy for women of childbearing age. This approach is particularly beneficial for those with MTHFR polymorphisms, a history of adverse pregnancy outcomes related to folate metabolism, or women wishing to avoid UMFA. Individual genetic, nutritional, and health factors should be considered, and consultation with a healthcare provider is advised. Further large-scale trials are needed to establish standardized guidelines for the active form of folate supplementation. Notably, while periconceptional folic acid supplementation is established to reduce the risk of NTDs, it remains unclear whether the active form of folate confers equivalent or superior protective effects. Therefore, more large-scale, long-period RCTs are necessary to evaluate the role of the active form of folate for different characteristics of women.

## 5. Conclusion

The analysis suggests that the active form of folate supplementation may be more beneficial than folic acid for women of childbearing age in terms of increasing plasma folate, increasing erythrocyte folate, and decreasing unmetabolized folic acid. It may also decrease the incidence of adverse pregnancy outcomes, and increase the subsequent pregnancy rates for women with adverse pregnant history. However, there was no significant effect of active form of folate on homocysteine, vitamin B_12_, and betaine as compared to folic acid. Large sample size and well-designed RCTs are needed to further investigate this topic.

## Author contributions

**Conceptualization:** Min Xie, Jing Zhang.

**Data curation:** Min Xie, Xuemei Qing.

**Investigation:** Min Xie, Hailong Huang, Jing Zhang.

**Methodology:** Min Xie, Xuemei Qing, Jing Zhang.

**Validation:** Min Xie, Xuemei Qing, Hailong Huang, Jing Zhang.

**Writing – original draft:** Min Xie.

**Writing – review & editing:** Min Xie, Xuemei Qing, Hailong Huang, Jing Zhang.

## Supplementary Material
