# Supplementary material for: The effectiveness and safety of the active form of folate on biochemical parameters in women of childbearing age: A systematic review and meta-analysis
Source: Medicine (Baltimore). 2025 Dec 12;104(50):e46564. doi: 10.1097/MD.0000000000046564 (PMC12708167; doi:10.1097/MD.0000000000046564)

**Search strategy**

**Source: Pubmed; searched on: January 1, 2024; results: 132**

(("tetrahydrofolates"[MeSH Terms] OR Tetrahydrofolates[Text Word] OR “5-methyltetrahydrofolate” [Text Word] OR 5-MTHF[Text Word] OR “5-methylTHF”[Text Word] OR methyltetrahydrofolic[Text Word] OR l-5-methyltetrahydrofolic[Text Word] OR 5-methyltetrahydrofolic[Text Word] OR methylfolate[Text Word] OR “methyl folate”[Text Word] OR methyltetrahydrofolate OR “methyl tetrahydrofolate” OR l-methylfolate OR l-5-methyltetrahydrofolate OR 6(s)-5methyltetrahydrofolate OR 5-methyltetrahydrofolic OR 6(s)-5-methyltetrahydrofolic OR “methylfolate*” OR “L-5-MTHF” OR “(6S)-5-methyltetrahydrofolic acid” OR “(6S)-5-MTHF” ) AND (“Reproductive age” OR “Pregnancy*” OR ”fertile*” OR “Childbearing” OR ”women”)) AND (Randomized Controlled Trial[ptyp] AND "humans"[MeSH Terms])

**Source: Cochrane; searched on: January 1, 2024; results: 126**

## 126 Trials matching (tetrahydrofolates OR Tetrahydrofolate OR 5methyltetrahydrofolate OR 5MTHF OR 5methylTHF OR methyltetrahydrofolic OR l5methyltetrahydrofolic OR 5methyltetrahydrofolic OR methylfolate OR methyl folate OR methyltetrahydrofolate OR methyl tetrahydrofolate OR lmethylfolate OR l5methyltetrahydrofolate OR 6(s)5methyltetrahydrofolate OR 5methyltetrahydrofolic OR 6(s)5methyltetrahydrofolic OR methylfolate* OR L5MTHF OR (6S)5methyltetrahydrofolic acid OR (6S)5MTHF ) in Title Abstract Keyword AND (Reproductive age OR Pregnancy* OR fertile* OR Childbearing OR women) in Title Abstract Keyword AND (randomized controlled trial or (trial* or random* or RCT*)) in Title Abstract Keyword - (Word variations have been searched)

**Source: Medline; searched on: January 1, 2024; results: 446**

"TX ( (tetrahydrofolates OR Tetrahydrofolate OR 5methyltetrahydrofolate OR 5MTHF OR 5methylTHF OR methyltetrahydrofolic OR l5methyltetrahydrofolic OR 5methyltetrahydrofolic OR methylfolate OR methyl folate OR methyltetrahydrofolate OR methyl tetrahydrofolate OR lmethylfolate OR l5methyltetrahydrofolate OR 6(s)5methyltetrahydrofolate OR 5methyltetrahydrofolic OR 6(s)5methyltetrahydrofolic OR methylfolate* OR L5MTHF OR (6S)5methyltetrahydrofolic acid OR (6S)5MTHF ) ) AND TX ( women or female or woman or females or Reproductive age OR Pregnancy* OR childbearing or fertile* ) AND TX ( rct or randomized control trial or randomized controlled trial or controlled trial or cohort or case control OR random* OR trial* )

**Source: Embase; searched on: January 1, 2024; results: 154**

1# tetrahydrofolates:ab,ti OR tetrahydrofolate:ab,ti OR 5methyltetrahydrofolate:ab,ti OR 5mthf:ab,ti OR 5methylthf:ab,ti OR methyltetrahydrofolic:ab,ti OR l5methyltetrahydrofolic:ab,ti OR methylfolate:ab,ti OR 'methyl folate':ab,ti OR methyltetrahydrofolate:ab,ti OR 'methyl tetrahydrofolate':ab,ti OR lmethylfolate:ab,ti OR l5methyltetrahydrofolate:ab,ti OR 6s5methyltetrahydrofolate:ab,ti OR 5methyltetrahydrofolic:ab,ti OR 6s5methyltetrahydrofolic:ab,ti OR methylfolate*:ab,ti OR l5mthf:ab,ti OR ‘6s5methyltetrahydrofolic acid':ab,ti OR 6s5mthf:ab,ti

2# women:ab,ti OR female:ab,ti OR woman:ab,ti OR females:ab,ti OR 'reproductive age':ab,ti OR pregnancy*:ab,ti OR childbearing:ab,ti OR fertile*:ab,ti

3# #1 AND #2

4# rct:ab,ti OR random*:ab,ti OR 'randomized controlled trial':ab,ti OR 'randomized control trial':ab,ti OR 'controlled trial':ab,ti OR trial*:ab,ti OR 'case control':ab,ti

5# #3 AND #4

**Search in Chinese: CNKI (China National Knowledge Infrastructure); results: 136**

SU = (huoxingyesuan OR siqingyesuan + siqingyesuangai + jiajisiqingyesuan + yajiajisiqingyesuan + '5-jiajisiqingyesuan')

AND ((yulingqi + yulingqifunv + yulingqinvxing) OR (yunqi + weiyunwei + yunqianjiyunqi + yunqifunv + yunqinajiyunqi + renshenqi) OR (buliangrenshen + buliangrenshenjieju))

**Search in Chinese:Wanfang Database; results: 240**

Zhuti: (huxingyesuan OR siqingyesuan OR siqinagyeusangai OR jiajisiqingyesuan OR yajijiasiqingyesuan OR '5-jiajisiqingyesuan' ) and Zhuti:(yulingqi OR yulingqifunv OR yulingqinvxing OR yunqi OR weiyunqi OR yunqianjiyunqi OR yunqifunv OR yunqianheyunqi OR renshenqi)

**Sensitivity analysis**

**Plasma folate**

| Study omitted | Estimate | [ 95% Conf. Interval ] | |  |
| --- | --- | --- | --- | --- |
| Cochrane 2023 | 2.2391951 | 1.1179634 | 4.4849362 |  |
| Diefenbach 2013 | 2.0373254 | .92526102 | 4.4859719 |  |
| Fohr 2002 | 1.3802352 | .95872444 | 1.9870665 |  |
| Giunta 2018 | 2.1286116 | 1.0435847 | 4.3417535 |  |
| Hekmatdoost 2015 | 1.844687 | .88242459 | 3.8562729 |  |
| Henderson 2018 | 2.0000327 | .96744353 | 4.1347432 |  |
| Houghton 2006 | 2.0483763 | .9957999 | 4.2135425 |  |
| Lamers 2006 | 2.0844171 | .99779409 | 4.3544006 |  |
| Venn 2002 | 2.1905 | 1.074923 | 4.4638453 |  |
| Combined | 1.9725221 | 1.0271509 | 3.7879957 |  |


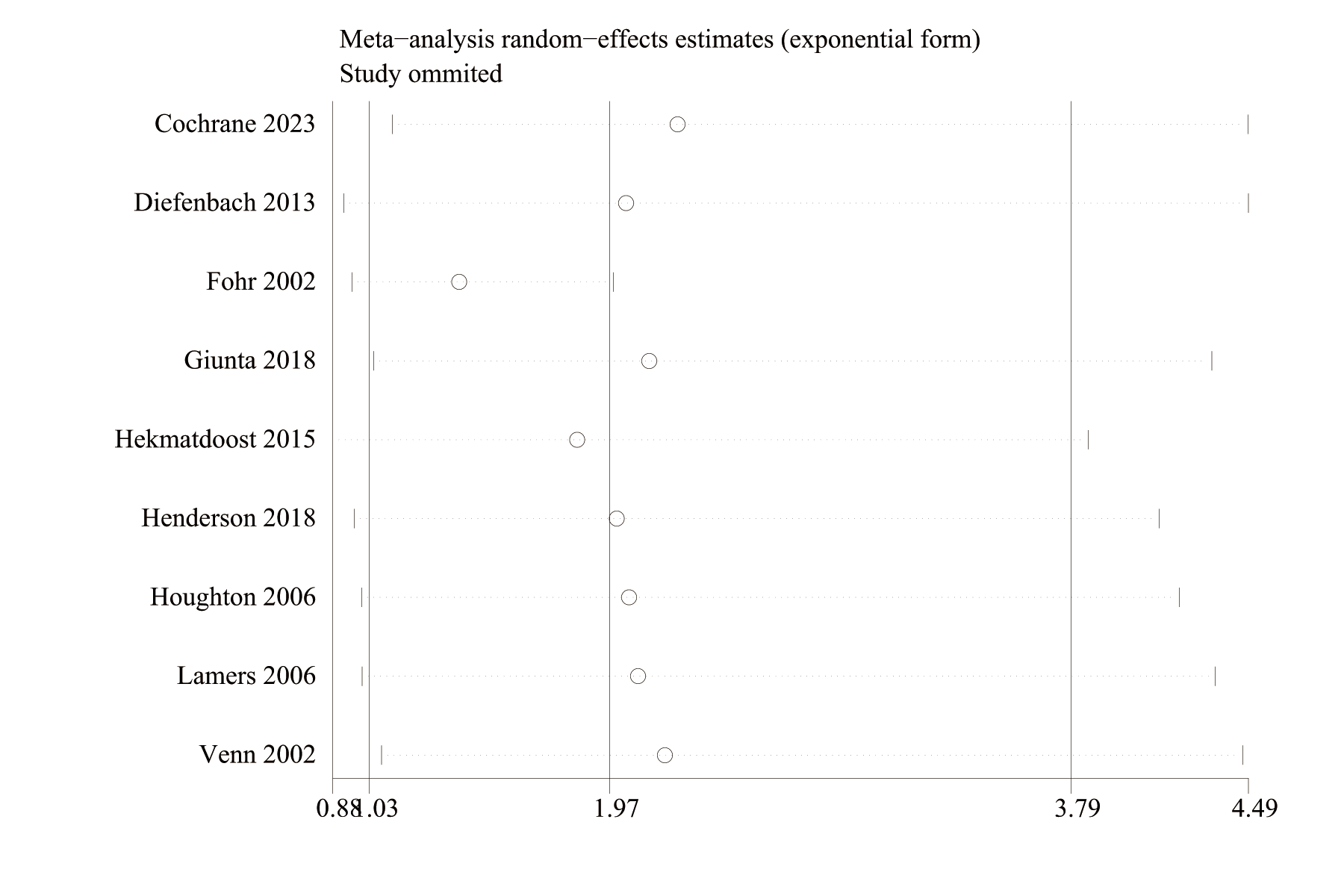


**erythrocyte folate**

| Study omitted | Estimate | [ 95% Conf. Interval ] | |  |
| --- | --- | --- | --- | --- |
| Cochrane 2023 | 1.6937871 | 1.1995705 | 2.3916183 |  |
| Diefenbach 2013 | 1.5024124 | .98312539 | 2.2959871 |  |
| Fohr 2002 | 1.6403819 | 1.1061816 | 2.4325597 |  |
| Henderson 2018 | 1.4007311 | .99423659 | 1.9734212 |  |
| Houghton 2006 | 1.4666399 | 1.0055398 | 2.1391819 |  |
| Lamers 2006 | 1.4251428 | .98869604 | 2.0542529 |  |
| Venn 2002 | 1.6726327 | 1.1604656 | 2.4108429 |  |
| Combined | 1.5378204 | 1.0903539 | 2.1689211 |  |


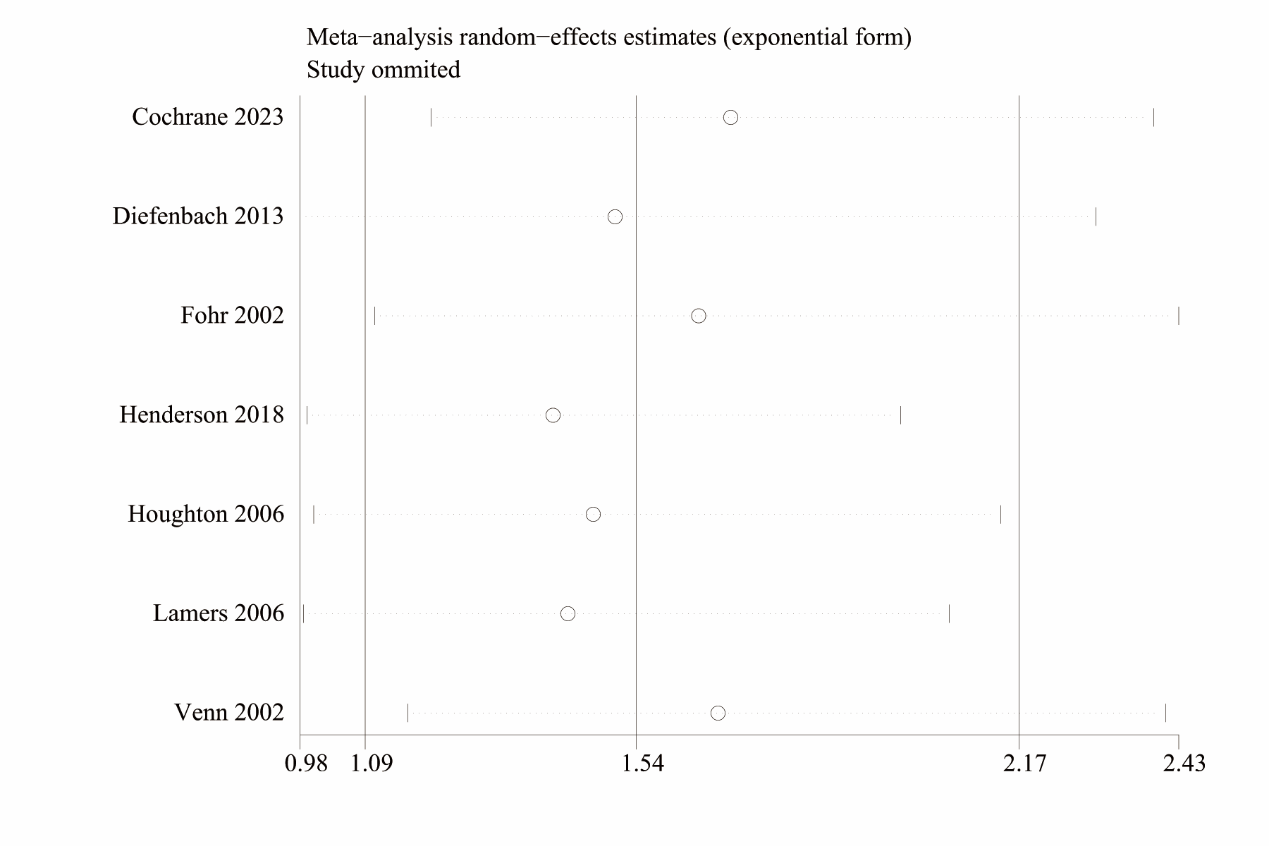

Supplement: Supplementary file 1 [file medi-104-e46564-s001.docx]
